# Comprehensive data of 10 fruit leaf classes captured for agricultural AI applications

**DOI:** 10.1016/j.dib.2025.111879

**Published:** 2025-07-10

**Authors:** Minhajul Abedin, Sujon Islam, Naznin Sultana

**Affiliations:** Department of Computer Science and Engineering, Daffodil International University, Daffodil Smart City, Birulia, Dhaka 1216, Bangladesh

**Keywords:** Fruit leaves, Dataset, Machine learning, Applications in agriculture, Image processing, Computer vision

## Abstract

This corpus contains 3173 high-quality images of leaves of ten commonly found fruit species in Bangladesh, namely Lotkon (306), Lychee (312), Mango (330), Black plum (304), Custard apple (304), Guava (325), Jackfruit (311), Aegle marmelos (336), Star Fruit (343), Plum (302). It is captured with Realme 7-Pro (64 MP primary camera) and Realme 8-Pro (108 MP primary camera) smartphones at nurseries near to Daffodil International University, Bangladesh. This dataset addresses the scarcity of high-quality, region-specific agricultural image datasets in South Asia, offering a unique combination of standardized smartphones-based imaging and controlled lighting to ensure consistant high-resolution visual data compared to existiong datasets. To ensure uniform image quality, all leaf specimens were photographed in controlled lighting against a white paper background. The dataset has a fairly balanced number of photos for each class, with between 300 and 343 photos for each class which makes it suitable for machine learning applications. To capture a complementary range of visual properties (leaf shape, venation patterns, edges, surfaces, etc.), the collection contains healthy leaf samples photographed from both the top and underside angels. This collection fulfills the need for South Asian region-specific datasets for agricultural images and could be utilized to develop computer vision applications, automated crops recognition systems, and agricultural monitoring software. The complete dataset is public and can be accessed on Mendeley Data repository and is hierarchically structured with separate directories for all the fruit species.

Specifications Table

**Table:** 

Subject	Biology,Computer Sciences
Specific subject area	Development of a fruit leaf classification dataset for machine learning and precision agriculture.
Type of data	**Table**: Summary of the number of images per class. **Image**: High-resolution fruit leaf photographs in JPG format. **Chart**: Class distribution of images. **Raw**: Original images collected from the nurseries.
Data collection	The dataset was obtained from the nurseries adjacent to Daffodil International University in Bangladesh. To ensure their image quality, leaves were hand-checked, washed, and photographed against a white paper background. The photographs have been taken using two smartphones Realme 7 Pro (64 MP primary camera) and Realme 8 Pro (108 MP primary camera). The original dataset consisted of high-resolution images(with dimensions such as 3000 × 4000 and 3456 × 4608 pixels).
Data source location	Geographical Location: Nurseries around Daffodil International University, Birulia, Savar, Dhaka-1216, Bangladesh. Storage Location: The dataset is hosted on Mendeley for public access.
Data accessibility	Repository name: Mendeley Data Data identification number: 10.17632/4gxzx6h7gv.2 Direct URL to data: https://data.mendeley.com/datasets/4gxzx6h7gv/2

## 1 Value of the Data

• The high variety within the dataset provides a strong foundation in terms of training and evaluation machine learning models with potential agricultural applications in hand.
• The process of the image acquisition holds a standardized protocol of controlled lighting and a uniformly colored white background to mitigate noise, leading to improved precision in model classification tasks.
• The dataset fills a gap in region-specific agricultural data by including images of ten most widely cultivated fruit species in Bangladesh, providing a valuable resource for research in precision farming, and automated crop monitoring.
• The structured directory organization of the dataset in addition to the public availability on Mendeley Data allows easy access and utilization of the dataset for training systems for AI-based plant identification.
• In addition, because standard smartphone cameras (Realme 7 Pro and Realme 8 Pro) have been used to capture the raw images and the processing of these images does not use high-quality setups, this dataset has potential to facilitate development of more affordable and mobile-based agricultural/AI solutions for farmers and agronomist-client.

## 2 Background

The necessity for extensive information from various geographic regions has been brought to light by recent developments in plant disease detection and agricultural monitoring [1,2]. In order to overcome the present shortcomings in region-specific agricultural data resources, we have compiled a dataset on ten fruit species that are often grown in Bangladesh. Successful smartphone-based data collection methods used in previous agricultural research [[3], [4], [5]] served as the basis for the methodological approach.

Realme 7 Pro and Realme 8 Pro smartphones with high-resolution cameras (64 MP and 108 MP main sensors, respectively) were used in the data gathering process to take pictures of leaves from nurseries near Daffodil International University. This strategy expands on new developments in agricultural surveillance via mobile devices [6,7]. Following established practices for developing plant image datasets, a white paper background was used for image capture [8,9].

To facilitate machine learning applications, the dataset structure keeps a balanced distribution of photos across species (300–343 images per category). With a particular focus on fruit species important to Bangladesh's agricultural industry, this methodical approach to data collecting builds upon already-existing agricultural imaging datasets [10,11].

Similar efforts inleaf classification using YOLO-based deep learning models have also been explored in the context of Bangladeshi plants [12] and cotton leaf deaction [13], reinforcing the viability of object detection techniques in agricultural imaging tasks.

## 3 Data Description

**Fig. 1:**
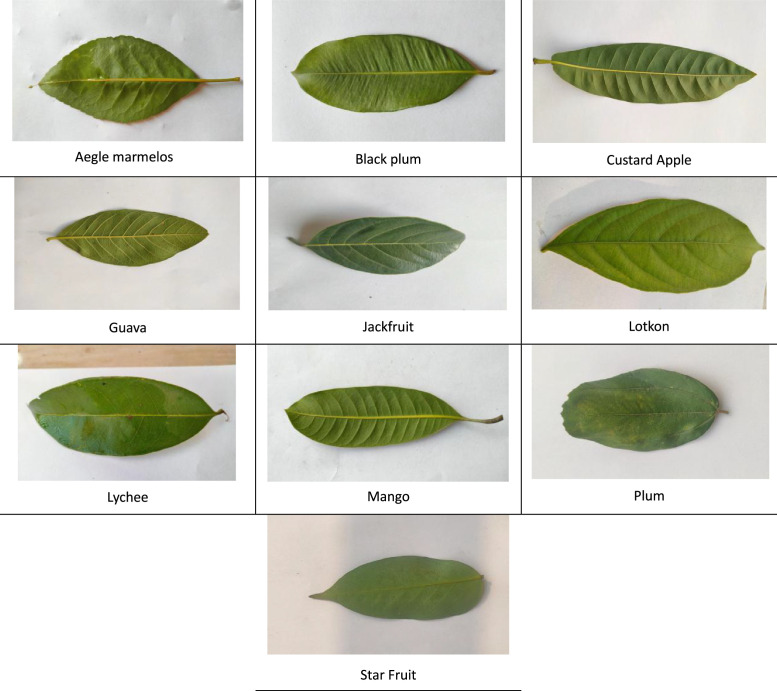
Images of ten different fruit species.

The collection consists of 3173 fruit leaf images spanning ten species, gathered from nurseries in the vicinity of Daffodil International University in Bangladesh. Fig. 1 shows distinct examples from each of the ten fruit species: Aegle marmelos, Black plum, Custard Apple, Guava, Jackfruit, Lotkon, Lychee, Mango, Plum, and Star Fruit and detailed description of class are provided separately in the subsection mentioned below. The images are organized based on fruit species which will help researchers to have a clear insight into the dataset.

### 3.1 Aegle marmelos

**Fig. 2:**
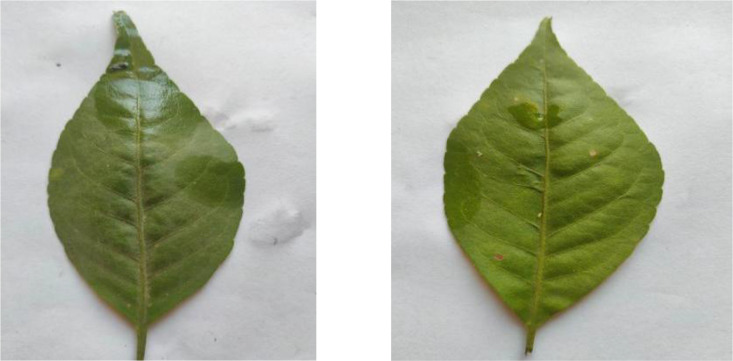
Image Of Aegle marmelos.

Fig. 2 shows Aegle marmelos leaves photographed against a white background. The leaves display their characteristic oval shape with pointed tips and serrated edges. These leaves are known for their medicinal properties and are significant in traditional medicine. The dataset contains 336 high-quality images showing various leaf specimens.

### 3.2 Black plum

**Fig. 3:**
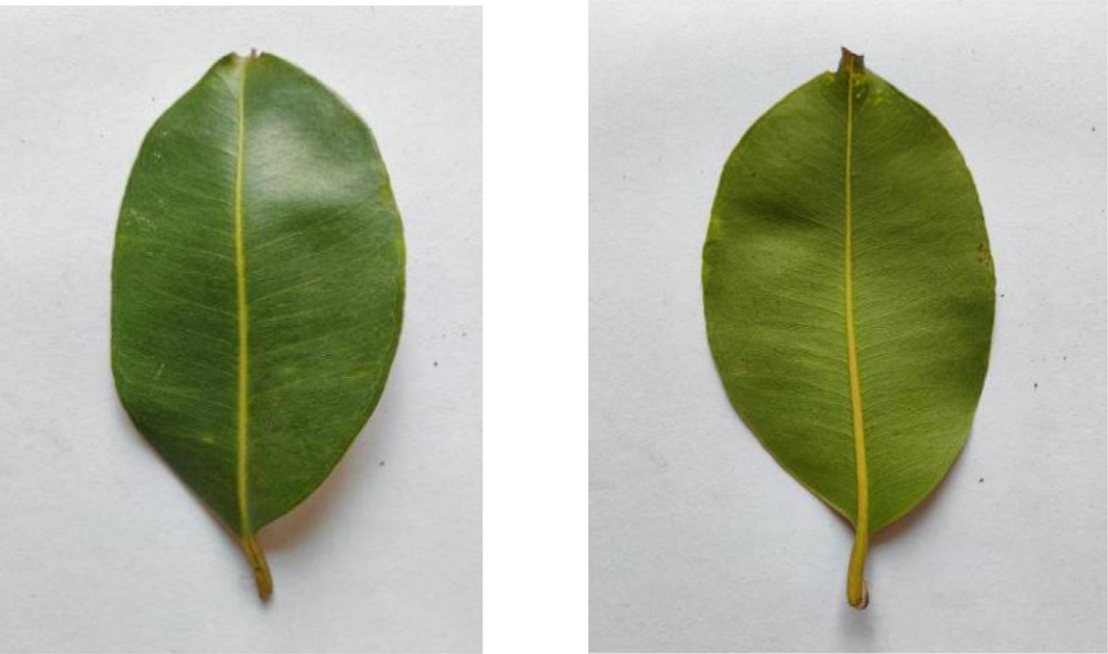
Image Of Black plum.

Black plum leaves, shown in Fig. 3, feature their distinctive elongated shape and smooth margins. The leaves exhibit a dark green glossy surface with prominent midribs, and each leaf measures approximately 5–10 cm in length. The dataset contains 304 images capturing different specimens against a standardized white backdrop.

### 3.3 Custard apple

**Fig. 4:**
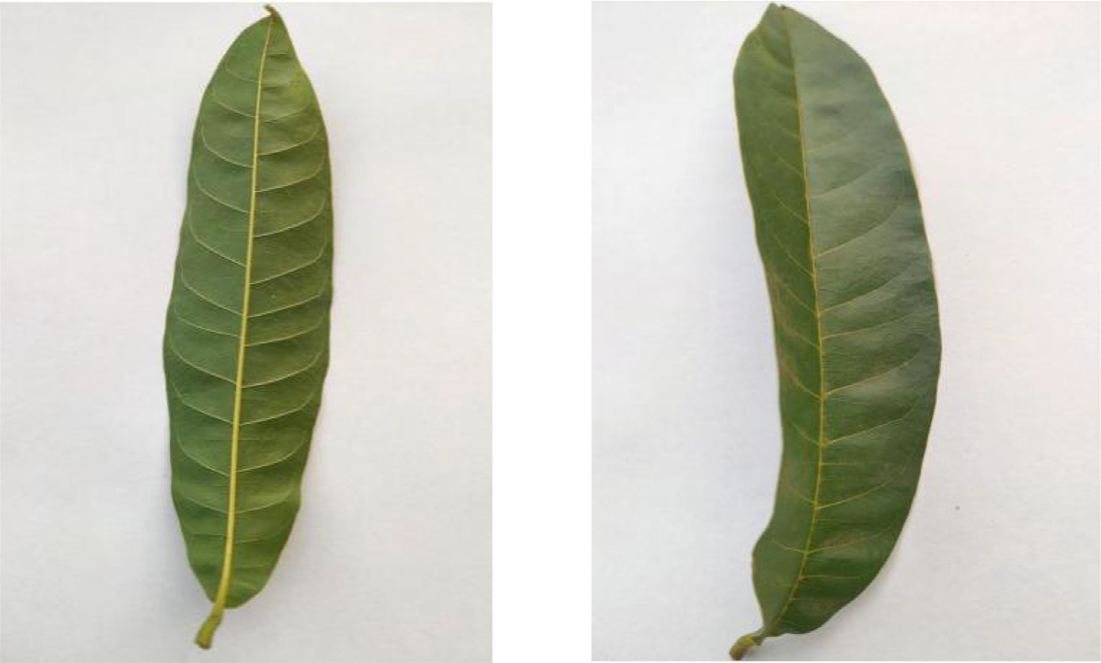
Image of custard apple.

The dataset includes 304 images of Custard Apple leaves, as shown in Fig. 4. The leaves exhibit their typical oblong shape with prominent veination patterns and a slightly wavy margin. These leaves are characterized by their soft texture and light to medium green coloration.

### 3.4 Guava

**Fig. 5:**
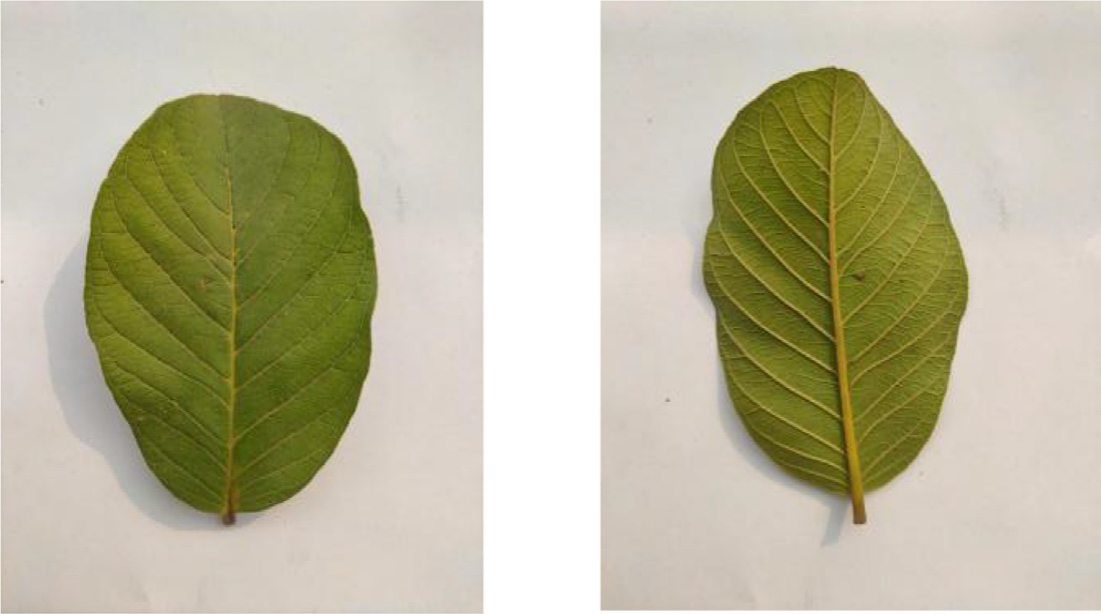
Image of guava.

Fig. 5 presents Guava leaf samples from the 325 images in this category. The leaves show their characteristic oval shape with visible parallel veins running from the midrib to the margins. The leaves have a leathery texture and are arranged opposite each other on the branches.

### 3.5 Jackfruit

**Fig. 6:**
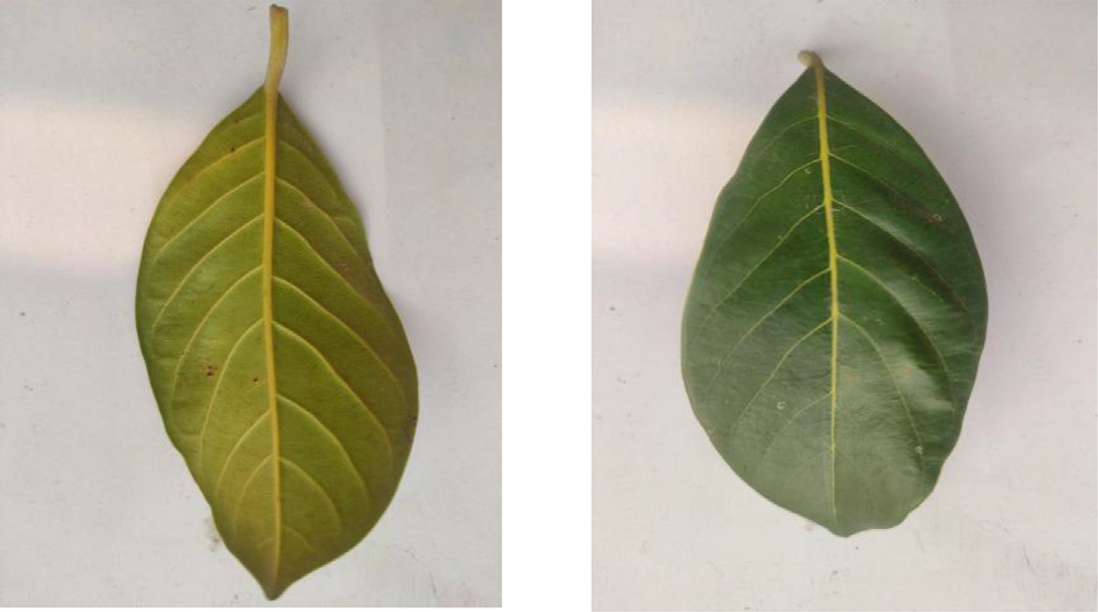
Image of jackfruit.

Jackfruit leaves, displayed in Fig. 6, comprise 311 images in the dataset. The leaves demonstrate their glossy appearance and robust structure with entire margins and a dark green upper surface. These large, leathery leaves are typical of tropical evergreen trees.

### 3.6 Lotkon

**Fig. 7:**
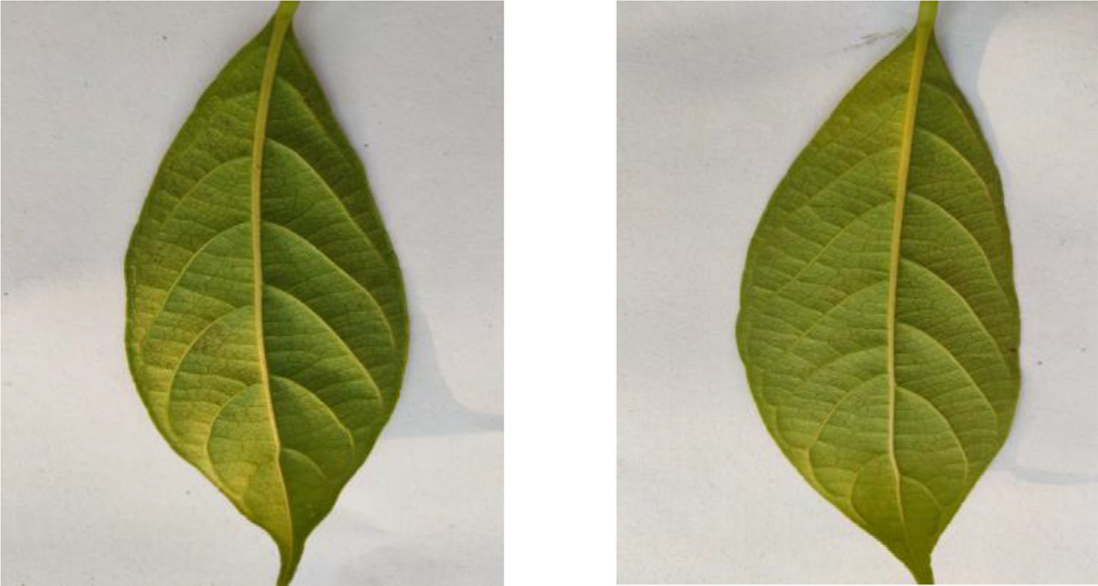
Image of lotkon.

The dataset contains 306 Lotkon leaf images, as shown in Fig. 7, capturing their distinctive leaf characteristics. The leaves are known for their smooth texture and elliptical shape with finely serrated margins. Each leaf displays a characteristic venation pattern typical of the species.

### 3.7 Lychee

**Fig. 8:**
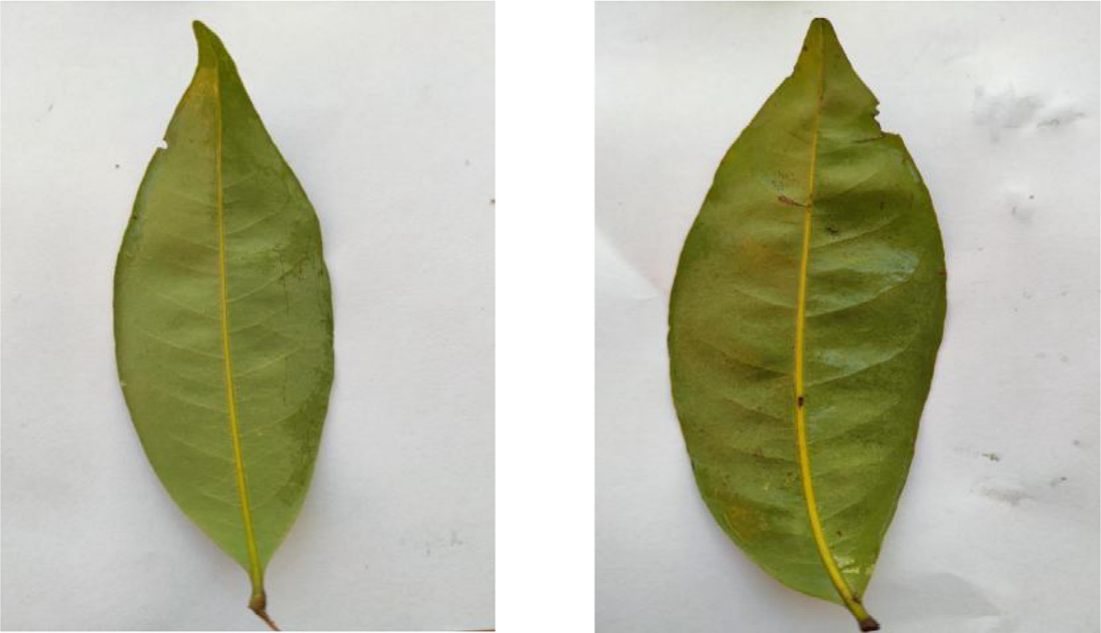
Image of lychee.

Fig. 8 presents examples from the 312 Lychee leaf images, showing their paired leaflet arrangement. Each leaflet exhibits a distinctive glossy appearance with a deep green hue, featuring a leathery surface texture, pointed tips, and smooth-edged margins.

### 3.8 Mango

**Fig. 9:**
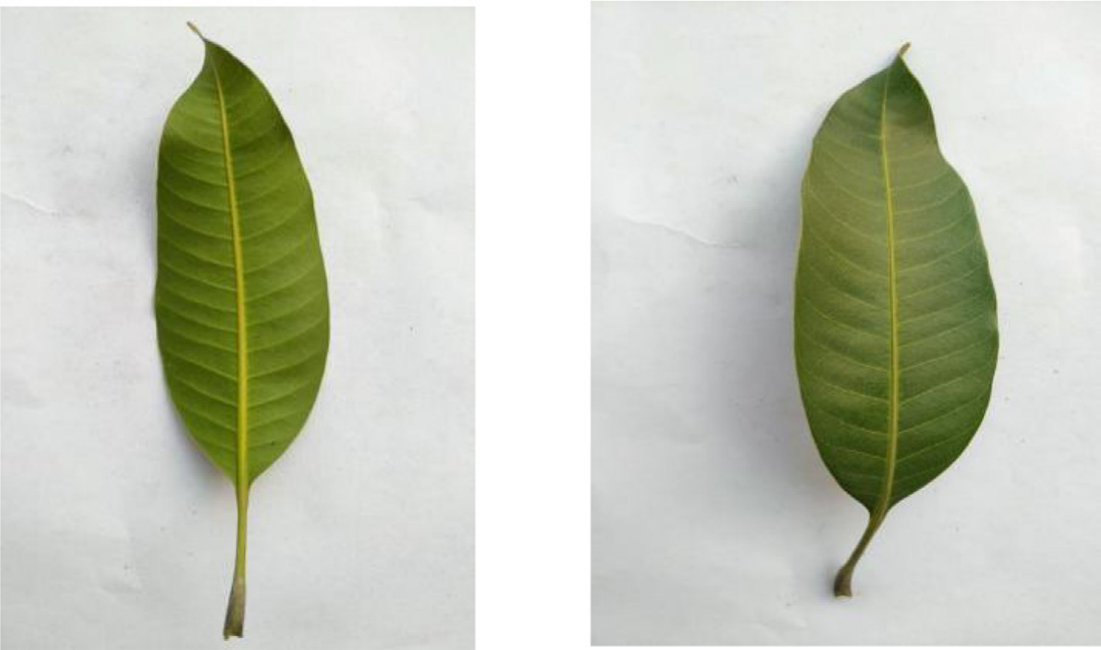
Image of mango.

The collection includes 330 Mango leaf images, as shown in Fig. 9, featuring their lanceolate shape and leathery texture. These leaves are distinguished by their long, narrow form and their tendency to be slightly curved, with smooth margins and a prominent midrib.

### 3.9 Plum

**Fig. 10:**
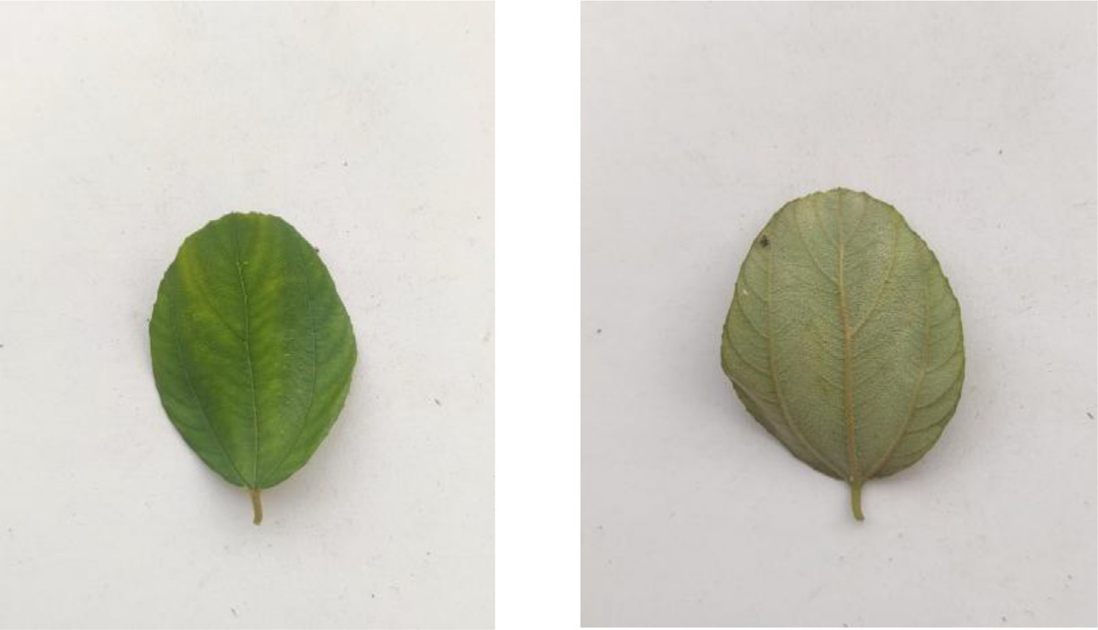
Image of plum.

Fig. 10 shows samples from the 302 Plum leaf images, displaying their ovate shape and serrated margins. The leaves feature a distinct network of veins and typically show a gradual tapering towards the tip with a broad base.

### 3.10 Star fruit

**Fig. 11:**
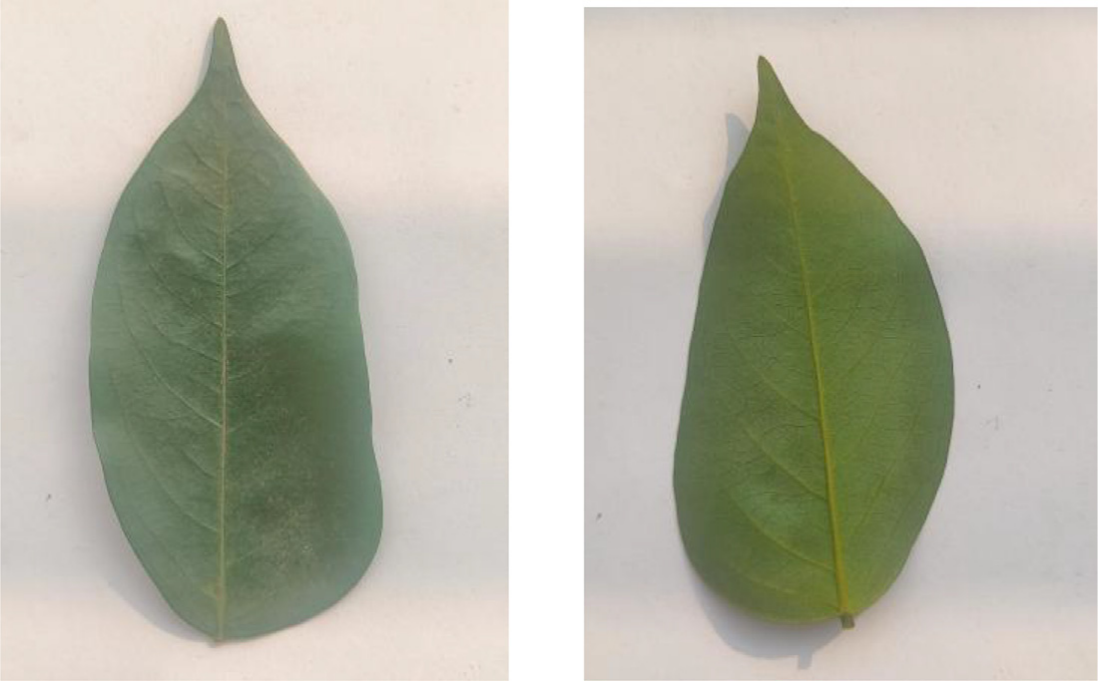
Image of star fruit.

The dataset contains 343 Star Fruit leaf images, as shown in Fig. 11, featuring their compound leaf structure. The leaves are characterized by their pinnate arrangement and smooth margins, with each leaflet showing a distinct midrib and secondary veins.

The image collection is organized within a primary directory titled 'Fruit_Leaves,' containing ten distinct sub-directories as detailed in Table 1. Consistency in image quality was maintained by capturing all specimens at a standardized resolution. The image are stored in JPG format with an average file size of approximately 2–4 MB per image. To optimize performance in machine and deep learning applications, the dataset features a carefully balanced number of images across all classes, with each category containing approximately 300–343 samples. This even distribution, as illustrated in Table 1, ensures minimal class bias and supports robust model training. All image are original captures without any augmentation techniques applied, ensuring authentic representation of each species.

**Table 1:** The number of leaves per class in our dataset.

Class Name	Number of Images	Image Format	Resolution
Aegle marmelos	336	JPG	High-Resolution
Black plum	304	JPG	High-Resolution
Custard Apple	304	JPG	High-Resolution
Guava	325	JPG	High-Resolution
Jackfruit	311	JPG	High-Resolution
Lotkon	306	JPG	High-Resolution
Lychee	312	JPG	High-Resolution
Mango	330	JPG	High-Resolution
Plum	302	JPG	High-Resolution
Star Fruit	343	JPG	High-Resolution
Total	3173		

## 4 Experimental Design, Materials and Methods

### 4.1 Data collection sites

Data collection took place at several nurseries near Daffodil International University. Because these sites were located close together it allowed for images to be collected frequently over the course of the collection period. We know the different fruit tree species that were growing in every nursery, and we have a collection of leaf samples from the data that were grown in each nursery. Collaborating with several nearby nurseries helped us build a diversified dataset while also managing logistics and travel time. Due to the proximity of the locations, our team could return to each location several times to collect samples under constant conditions.

**Fig. 12:**
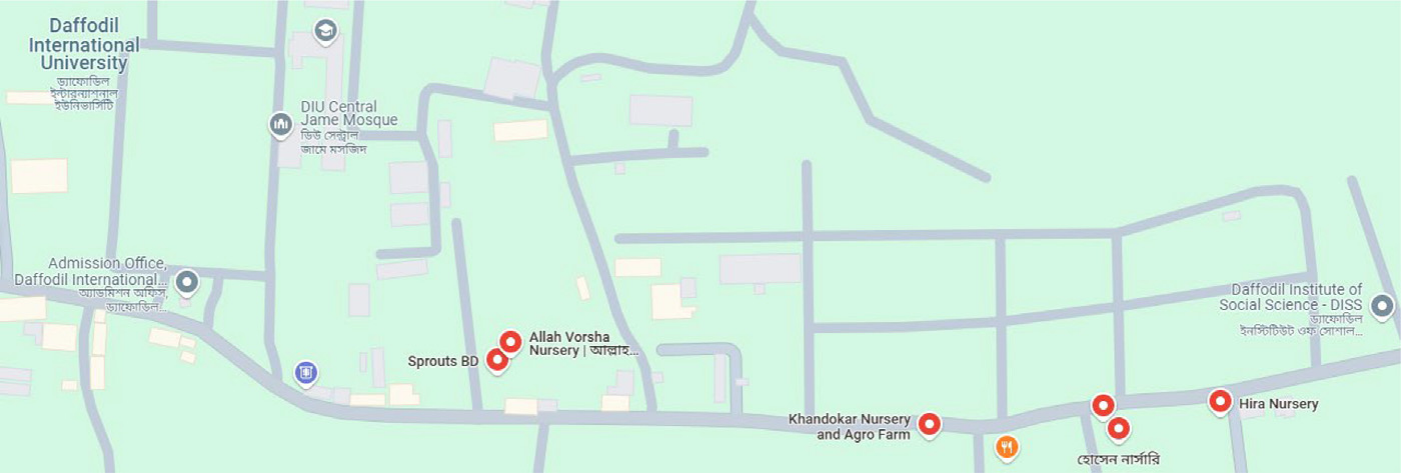
Image of the map.

**Fig. 13:**
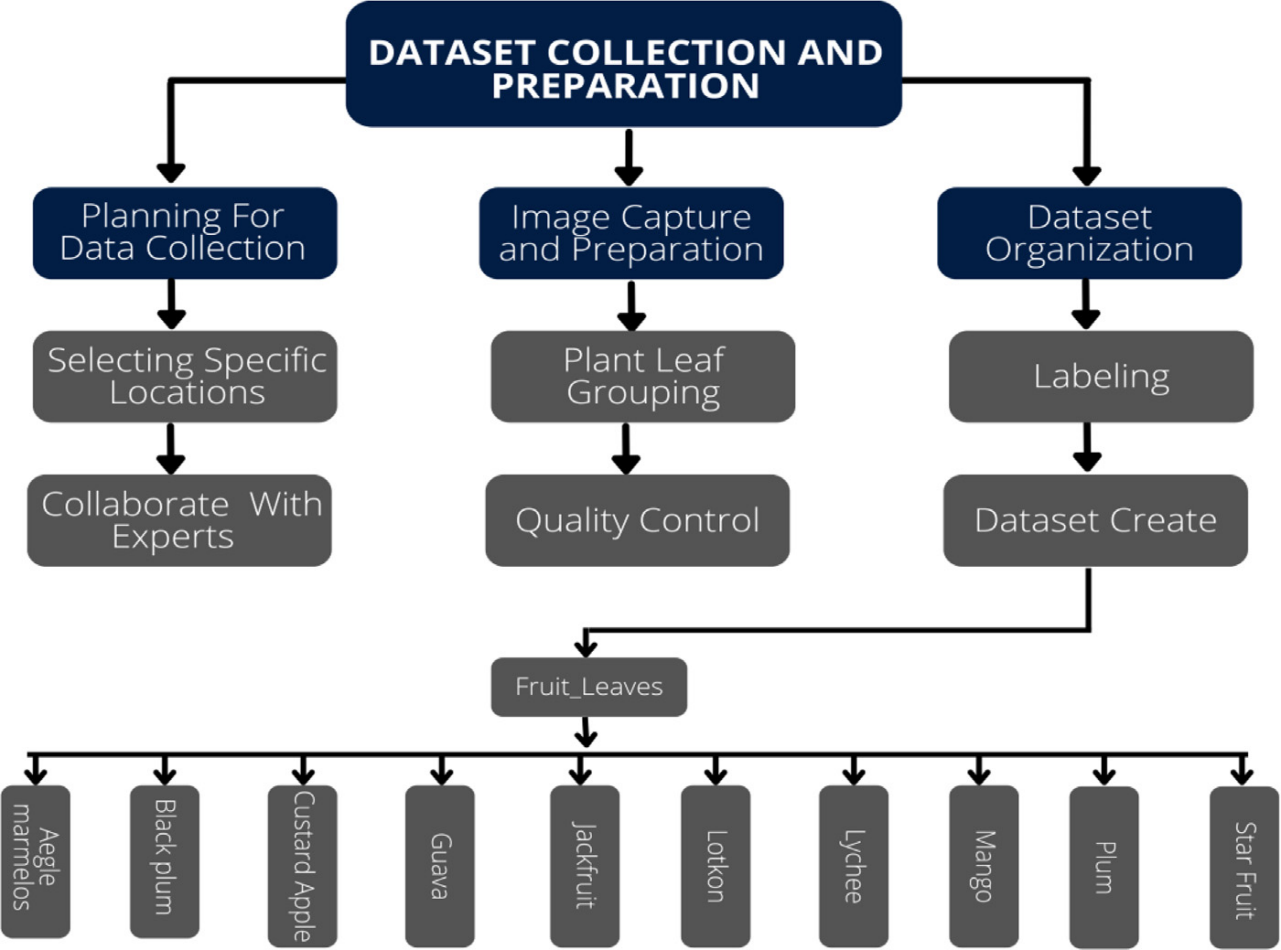
Overall working procedure.

Here are the locations on our university campus from which we collected our leaf samples (shown on the below map) (Fig. 12, Fig. 13):

With the help of some very knowledgeable nursery employees who helped us identify good specimens, we spent a lot of time examining each fruit leaf variety and what set them apart from each other. Their knowledge of local fruit trees meant they could spot healthy leaves and identify minute species variations. To ensure we acquired the most representative samples for each species, the specialists helped us develop a methodical strategy for leaf selection. Their counsel allowed us to establish reliable collection procedures against which we could ensure consistency in the effort. Collaborating with regional experts, our team compiled a comprehensive dataset that accurately represents the characteristic samples for every fruits species.

### 4.2 Mobile camera details

We took pictures of the leaves with Realme 7 Pro and Realme 8 Pro smartphone models. The specimens were arranged on a concentration of white paper, and to prevent long exposure images and false color representation, the lighting was kept the same for all of specimens. By carefully choosing the photography conditions, we were able to retain very good image quality throughout the collection. The cameras of both smartphones are pretty advanced and the specific specs of both can be seen in Table 2.

**Table 2:** Detailed specifications outline.

Feature	Realme 7 Pro	Realme 8 Pro
Primary Sensor	64MP, f/1.8, Sony IMX682, 1/1.73″, 0.8 µm, PDAF	108MP, f/1.89, Samsung HM2, 1/1.52″, 0.7 µm, PDAF
Ultrawide Sensor	8MP, f/2.3, 119°FOV, 1/4.0″, 1.12µm	8MP, f/2.3, 119°FOV, 1/4.0″, 1.12µm
Focal Length	26 mm (wide)	26 mm (wide)
ISO Range	100–6400	100–6400

Each image in our collection went through strict quality checks to meet specific standards we set for sharpness, lighting conditions and image that did not meet focus quality standards was discarded. Through this detailed screening process, we created a complete and reliable dataset of fruit leaf images. The dataset generated can be used to build new tools for plant identification and classification techniques by researchers.

## Limitations

Users should be aware that our fruit leaf collection has limits, just like any other research data. During the data collection process, we encountered a number of real-world difficulties, such as limitations pertaining to our techniques, the surrounding environment, and the camera gear. Other researchers will be better able to exploit this information and identify areas for future development if these restrictions are made known. The primary restrictions that we found are as follows:

1. Location Restrictions: In Bangladesh, we collected all of the leaf samples from nurseries close to our institution. Due to its narrow geographic focus, our dataset may not accurately represent the appearance of these fruit leaves in various locales or climates.
2. Lab-like Setting: Although it improves image clarity, our decision to take pictures of leaves on white backgrounds with regulated lighting isn't representative of actual farm circumstances. In real fields, leaves emerge against a variety of backgrounds under shifting environmental and light conditions.
3. Seasonal Gaps: Neither the growing cycle nor the seasonal variations in leaves are depicted in the dataset. The look and characteristics of leaves can be slightly changed by these modifications.
4. Emphasis on Healthy Leaves: We have excluded significant differences in the appearance of diseased, injured, or stressed leaves by exclusively gathering samples of healthy leaves. As a result, the dataset is less valuable for creating instruments to identify plant issues. Future expansions of the datasets are planned to include leaf samples from diverse regions of Bangladesh, as well as images capturing seasonal variations and diseased leaves.

## Ethics Statement

Our work meets all ethical guidelines required by Data in Brief journal. We didn't work with human participants, conduct animal testing, or use any social media data. During leaf collection, we took care to avoid harming plants or disturbing the nursery environment. All samples were gathered with proper permission from nursery owners and with approval from the Department of Computer Science and engineering at Daffodil International University.

## Credit Author Statement

**Minhajul Abedin:** Conceptualization, Methodology, Project Administration, Data Collection, Writing. **Sujon Islam:** Data Collection, Investigation, Writing, Methodology, Visualization. **Naznin Sultana:** Supervision, Writing - Review & Editing, Formal Analysis.

## Data Availability

Mendeley DataMulti-Class Fruit Leaf Classification Dataset (10 Classes) (Original data). The dataset is organized in a primary directory named “Fruits_Leaves”, with ten sub-directories, each named after a fruit species for easy identification and access. While the images within these class folders aren’t named serially, this shouldn’t be an issue for classification tasks, as their organization by class is the key factor.
